# Fabrication of Cell-Laden Hydrogel Fibers with Controllable Diameters

**DOI:** 10.3390/mi8050161

**Published:** 2017-05-18

**Authors:** Zhuoqun Cheng, Maosheng Cui, Yu Shi, Yanding Qin, Xin Zhao

**Affiliations:** 1 Institute of Robotics and Automatic Information System, Nankai University, Tianjin 300350, China; nkzhuoqun@mail.nankai.edu.cn (Z.C.); 2120160463@mail.nankai.edu.cn (Y.S.); 2 Tianjin Key Laboratory of Intelligent Robotics, Nankai University, Tianjin 300350, China; 3 Institute of Animal Sciences, Tianjin 300312, China; tjxmcui2014@126.com

**Keywords:** hydrogel fibers, controllable fabrication, cell-laden, pneumatic injection

## Abstract

Cell-laden hydrogel fibers are widely used as the fundamental building blocks to fabricate more complex functional three-dimensional (3D) structures that could mimic biological tissues. The control on the diameter of the hydrogel fibers is important so as to precisely construct structures in the above 3D bio-fabrication. In this paper, a pneumatic-actuated micro-extrusion system is developed to produce hydrogel fibers based on the crosslinking behavior of sodium alginate with calcium ions. Excellent uniformity has been obtained in the diameters of the fabricated hydrogel fibers as a proportional-integral-derivative (PID) control algorithm is applied on the driving pressure control. More importantly, a linear relationship has been obtained between the diameter of hydrogel fiber and the driving pressure. With the help of the identified linear model, we can precisely control the diameter of the hydrogel fiber via the control of the driving pressure. The differences between the measured and designed diameters are within ±2.5%. Finally, the influence of the calcium ions on the viability of the encapsulated cells is also investigated by immersing the cell-laden hydrogel fibers into the CaCl_2_ bath for different periods of time. LIVE/DEAD assays show that there is little difference among the cell viabilities in each sample. Therefore, the calcium ions utilized in the fabrication process have no impact on the cells encapsulated in the hydrogel fiber. Experimental results also show that the cell viability is 83 ± 2% for each sample after 24 h of culturing.

## 1. Introduction

Three-dimensional bio-fabrication has been extensively investigated in recent years [1,2,3]. Through a bottom-up implementation strategy and using cell-seeding materials, one can fabricate complex three-dimensional (3D) functional personalized tissue structures [4,5,6]. For instance, some organs or tissues have been fabricated and used in the clinic application, such as the auricule, bone, skin, and the cartilage tissue of nose [7,8,9,10].

One typical and popular strategy of bio-fabrication is bio-printing, i.e., the layer by layer coating of cell-laden bio-ink. Significant achievements have been made in this area. However, this process is confronted with the difficulty of achieving a good balance between the conditions for printing highly viable cells and producing sufficiently strong scaffold to support clinical scale cell-laden structures at the same time [11]. To overcome this challenge, several other bio-fabrication approaches have also been developed [12,13,14]. One more practical approach is the preparation and assembly of cell-laden hydrogel fibers into more complex structures [15,16]. This method separates the encapsulating of cells into the bio-compatible ‘blocks’, i.e., hydrogel fibers, and the constructing of 3D tissues using these ‘cell-laden blocks’. Therefore, the damage to the cells during the bio-fabrication can be reduced and the mechanical strength of the fabricated substrates can be improved [17,18]. In this method, the size control of the hydrogel fibers becomes very important as it determines the geometric resolution of the fabricated structures.

Great efforts have been made to achieve the controllable preparation of the hydrogel fibers [19,20,21]. Yu and Andrea [22] found a qualitative relationship between the diameter of the hydrogel fiber and the trajectory velocity through the experimental results. It is still difficult to control the diameter of the hydrogel fiber due to a lack of quantitative relationship. Khalil and Sun [23] proposed a mathematic model for the diameter control of the hydrogel during the fabrication. However, this mathematic model misses some important parameters, such as the solution viscosity and the nozzle diameter. A concise and effective method is desirable to precisely predict and control the diameter through some typical parameters during the fabrication of hydrogel fibers.

In this paper, a pneumatic micro-extrusion system with controllable driving pressure is developed for the fabrication of Ca-Alginate hydrogel fibers. The sodium alginate solution can be extruded out through a tapered nozzle and falls into the CaCl_2_ solution by adjusting the driving pressure. The hydrogel fibers can then be fabricated as a result of the crosslinking effect. A proportional-integral-derivative (PID) control algorithm is applied in the pressure control to improve the precision on the driving pressure control. A series of experiments are carried out and a good uniformity is observed in the fabricated hydrogel fibers. The experiment results also show a good linear relationship between the diameter of the hydrogel fiber and the driving pressure. Linear curve fitting is then utilized to identify the linear model for each nozzle. The linear model is then utilized to control the diameters of the fabricated hydrogel fibers. In the cell viability test, porcine fetal muscle fibroblast cells are chosen to fabricate the cell-laden hydrogel fibers.

## 2. Materials and Methods

### 2.1. Materials

The sodium alginate solution is prepared by mixing sodium alginate (LF10/60, FMC Biopolymer, Drammen, Norway) with deionized water at a concentration of 30 mg∙mL^−1^. As the sodium alginate solution is viscous and air is often mixed into the solution to form bubbles, the solution can be used in the experiments after a standing time of 15 min. The CaCl_2_ (Sigma Aldrich, St. Louis, MO, USA) crosslinker solution is prepared at a concentration of 7 mg∙mL^−1^.

Porcine fetal muscle fibroblast cells are cultured in Dulbecco’s modified Eagle medium (DMEM, Gibco, CA, USA) with 10% fetal bovine serum (FBS, Gibco, CA, USA) and containing 10,000 units of 0.0065 mg∙mL^−1^ penicillin (Sigma Aldrich, St. Louis, MO, USA) and 0.05 mg∙mL^−1^ streptomycin (Sigma Aldrich, St. Louis, MO, USA) at 37 °C under 5% CO_2_ and 95% atmospheric air. After cells proliferate and cover 75% of the bottom of the culture dish, they are detached with 0.25% trypsin (Sigma Aldrich, St. Louis, MO, USA) for 3 min at 37 °C. While the adherent cells were completely suspended, the cell suspension is transferred to a 15 mL centrifuge tube and centrifuged at 800 g for 10 min so that we can obtain high-concentration cell solution. This cell solution is mixed in sodium alginate solution at a concentration of ~2 × 10^6^ cells∙mL^−1^.

### 2.2. Methods

#### 2.2.1. The Pneumatic Micro-Extrusion System

**Figure 1:**
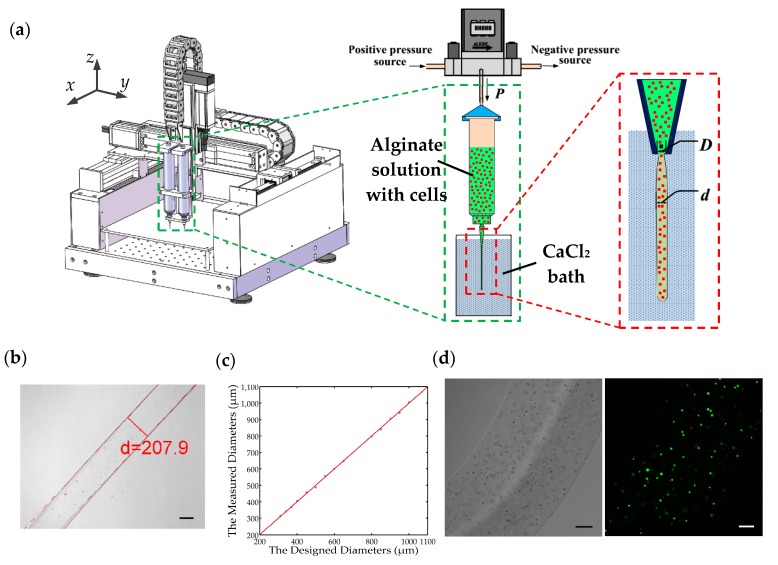
(**a**) Schematic diagram of the pneumatic micro-extrusion system: *P* indicates the driving pressure, *D* indicates the inner diameter of the nozzle, and *d* is the diameter of the hydrogel fiber; (**b**) calcium alginate fiber generated using a 0.2 mm nozzle with a driving pressure of 2.0 PSI, and its diameter measurement. The scale bar is 100 μm; (**c**) the comparison of the designed and measured diameters of 15 hydrogel fibers. The red line is the 45° line that represents a unitary mapping between the designed and measured diameters in the ideal case; (**d**) porcine fetal muscle fibroblast cell-laden calcium alginate fiber and the corresponding fluorescent image. The scale bar is 100 μm.

Figure 1a schematically illustrates the developed pneumatic micro-extrusion system. A dual valve pressure controller (PCD series, Alicat Scientific, Tucson, AZ, USA) is connected to a nitrogen cylinder (positive pressure source) and a vacuum pump (negative pressure source) to provide a continuous and controllable driving pressure to a pneumatic syringe (Nordson EFD, East Providence, RI, USA). The syringe is clamped to the worktable of a gantry robot with a workspace of 100 × 100 × 100 mm^3^ and a positioning repeatability of ±0.02 mm. Four tapered nozzles (Nordson EFD, East Providence, RI, USA) with commercially available tip inner diameter of 0.2, 0.25, 0.41, and 0.61 mm, are utilized throughout in this paper. The control program and user interface for the pneumatic micro-extrusion system is developed in the environment of LabVIEW (Version 2014 SP1, National Instruments, Austin, TE, USA).

A PID closed-loop control algorithm is applied in the driving pressure control. Compared with the open-loop control, the rise time of the system is reduced to 100 ms in closed-loop control, enabling fast response capability. Meanwhile, the steady state error has also been reduced to the noise level of the system. Consequently, the pneumatic micro-extrusion system with the PID closed-loop control can provide very stable control on the driving pressure during the fabrication of the Ca-Alginate hydrogel fibers.

#### 2.2.2. Fabrication of Ca-Alginate Hydrogel Fibers

The fabrication of the Ca-Alginate hydrogel fiber consists of four steps (an illustration video is provided in Supplementary Material), as listed below:

Step 1 The pre-prepared alginate solution is loaded into a 10 mL pneumatic syringe and the CaCl_2_ bath is placed under the nozzle. After a positive pressure signal is sent to the controller via the computer, the alginate solution begins to be extruded out from the nozzle.
Step 2 The motor in the *z*-axis direction receives a downward signal to dip the nozzle into the CaCl_2_ bath.
Step 3 The alginate solution is immediately exposed to the calcium ions in the cross-linker bath once it flows out from the nozzle. The crosslinking reaction occurs at the alginate-CaCl_2_ interface owing to the effect of the calcium ions. As a result, the alginate solution flow starts to solidify and turns into a calcium-alginate hydrogel fiber.
Step 4 When the fabrication is completed, the nozzle will be lifted out from the CaCl_2_ bath by the *z*-axis motor and a small negative pressure is given to balance the gravity of the alginate solution.

The Figure 1b shows the Ca-alginate hydrogel fiber fabricated using the 0.2 mm nozzle with a driving pressure of 2.0 PSI, and the hydrogel fiber will be collected in a Petri dish and imaged under a Nikon microscope (Nikon, Tokyo, Japan). We take microscopic images of 10 different parts of the hydrogel fiber and respectively measure their diameters using an in-house built MATLAB (Version 2014a, Mathworks, Natick, MA, USA) code. Then an average value of these 10 measurements is regarded as the diameter of the hydrogel fiber.

#### 2.2.3. Cell-Laden Alginate Hydrogel Fibers Preparation

The cells are evenly mixed into the sodium alginate solution before the preparation of cell-laden alginate hydrogel fiber. Then cell-laden hydrogel fibers can be fabricated using the same method described in Section 2.2.2. Along with the crosslinking of the alginate solution, the cells will be encapsulated in alginate hydrogel, which serves as the support for the cells. Consequently, the cell-laden alginate hydrogel fibers will be fabricated. There are many nanoscale pores in the hydrogel and the nutrients can transfer through them when the cell-laden hydrogel fibers are cultured in the cell culture medium. Figure 1d shows the bright-field and fluorescent images of a cell-laden hydrogel fiber after a 24-h culturing.

#### 2.2.4. The LIVE/DEAD Assay

The fluorescein diacetate (FDA) powder and propidium iodide (PI) powder are purchased from Sigma Aldrich. 5 mg of FDA powder is dissolved in 1 mL acetone and 0.1 mg of PI power is dissolved in 1 mL phosphate buffered saline (PBS), which serve as the stock solution. The LIVE/DEAD assay reagent is the mixture of 10 μL FDA solution and 5 μL PI solution diluted in the 1 mL PBS. With the help of ultraviolet laser, the live and dead cells are marked as green and red, respectively. The fluorescent image shown in Figure 1d is obtained by superimposing the 2D layer-scanned images in the *z*-axis under a confocal microscopy (Leica, Wetzlar, Germany). The cell viability can be calculated via counting the number of green and red dots in each layer-scanned image.

## 3. Results

### 3.1. The Fabrication of Hydrogel Fibers

**Figure 2:**
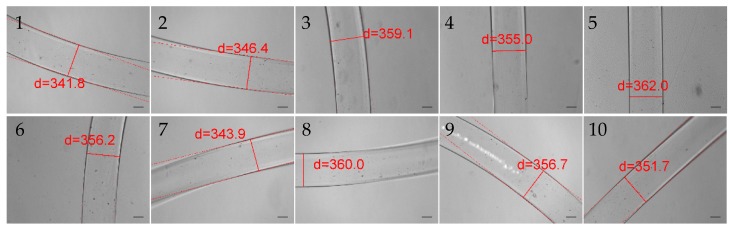
The measured diameters at the 10 positions of the hydrogel fiber fabricated in the configuration of (0.25, 1.5). The scale bar is 100 μm.

In the pneumatic micro-extrusion system, two parameters are controllable: the inner diameter of the nozzle *D* (unit: mm) and the driving pressure *P* (unit: PSI). Then we can use a pair (*D*, *P*) to represent the parameter configuration of the system. Four hydrogel fibers (each has a length over 20 cm) are fabricated in the following four different configurations: (0.2, 2.0), (0.25, 1.5), (0.41, 1.5), and (0.61, 1.0). For each sample, we measure the diameters at 10 different positions along the length. The measurements are implemented using the microscopy images obtained by the Nikon microscope. Figure 2 shows the 10 measured diameters of the hydrogel fibers fabricated in the configuration of (0.25, 1.5). All the measured diameters are recorded in Table 1. The diameters of the samples are 211.4 ± 8.3, 351.7 ± 8.3, 529.0 ± 7.8, and 785.2 ± 14.8 μm (mean ± s.d.), respectively. The experimental errors fall in the range of ±5% and the standard deviation of hydrogel fiber diameters of each group is less than 4%. This demonstrates that the diameter of the hydrogel fiber has a good uniformity fabricated in one experiment using a given configuration. In the meantime, it is also a good evidence that the developed pneumatic micro-extrusion system provides a stable platform to fabricate hydrogel fibers.

Further, we investigate the relationship between the diameter of the hydrogel fiber and the driving pressure by varying the driving pressure from 1.0 to 3.0 PSI with an interval of 0.4 PSI for a given nozzle. There are six parameter configurations for each nozzle, and we fabricate 10 hydrogel fibers in each configuration. The four nozzles previously described in Section 2.2.1 are employed and there are totally 24 parameter configurations. The diameters of the hydrogel fibers in these groups are measured and the statistics (the mean ± s.d.) are listed in Table 2. The standard deviations of each configuration are less than 2%. This result again shows the good uniformity of the diameters of the hydrogel fibers fabricated in the different experiments using the same configuration.

**Figure 3:**
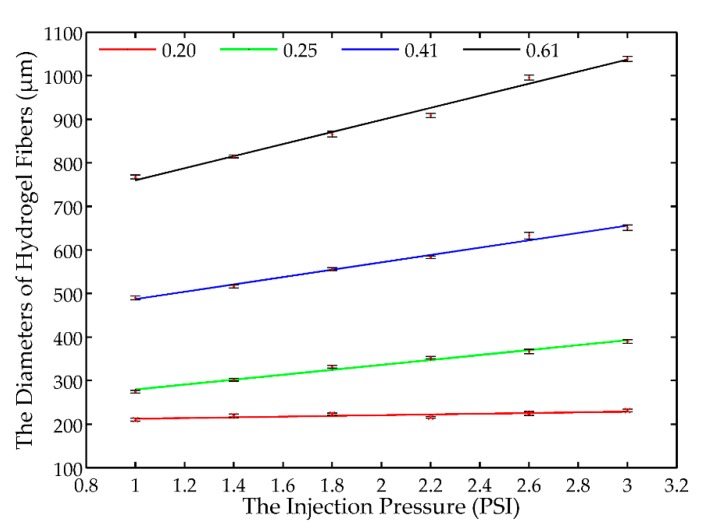
The measured diameters of the hydrogel fibers fabricated using four different nozzles and the corresponding linear fitting results with *r*-square values of 0.7, 0.991, 0.994, and 0.992, respectively.

The experimental results in Table 2 are also plotted in Figure 3, where the red dots with error bars indicate the means of the measured diameters of the hydrogel fibers. Interestingly, it is found that there is a linear relationship between the diameter of hydrogel fiber and the driving pressure for a given nozzle. Suppose the linear relationship between the driving pressure and the diameter of the hydrogel fiber can be expressed in the following formulation:

*d* = *kP* + *c* (1)

where *k* and *c* are the slope and offset of the linear relationship, respectively.

Linear curve fitting is then implemented and the identified linear models are also provided in Figure 3, and the identified coefficients are listed in Table 3. The *r*-square values of the fitted curve are above 0.99 except for the 0.2 mm nozzle with 0.7. Great agreements have been achieved between the measurements and the model predictions for 0.25, 0.41, and 0.61 mm nozzle and the linear relationship for 0.2 mm nozzle is not significant. Meanwhile, in the second row of Table 2, the measured diameters changes a little as the driving pressure increases, which indicates the diameter of hydrogel fibers fabricated using the 0.2 mm nozzle is not sensitive to the driving pressure. Therefore, the 0.2 mm nozzle will not be utilized in the following experiments. In addition, it is noted that *k* and *c* increase almost linearly with the inner diameter of nozzle *D*, and *c* is very close to *D*. These characteristics of the identified linear models indicate that *D* determines the minimum value of the diameter and the sensitivity of the diameter on the driving pressure. Consequently, it can serve as a basis to select a proper nozzle prior to the fabrication of hydrogel fibers.

**Figure 4:**
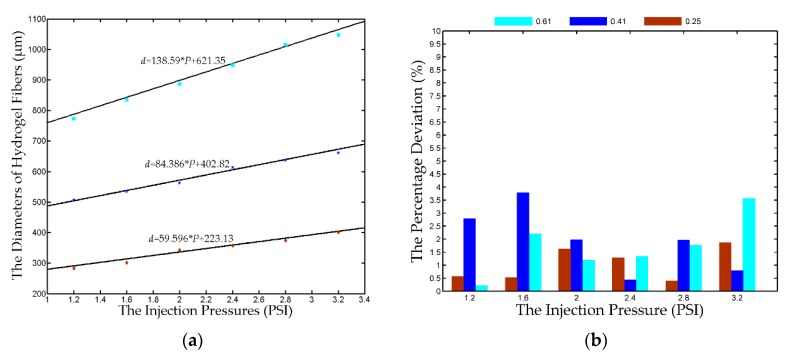
The comparison of the predicted and measured diameters. (**a**) The solid lines are drawn based on the identified linear models and the colored markers represent the experimental results; (**b**) the percentage deviation between the predicted and measured diameters. The legends with different colors represent the different inner diameters of the nozzles utilized in the fabrication of hydrogel fibers, respectively.

Experiments are further conducted to predict the diameter of the hydrogel fiber according to the identified linear models. Three nozzles (*D* = 0.25, 0.41, and 0.61 mm) are utilized and the driving pressures are set to 1.2–3.2 PSI with an interval of 0.4 PSI. The same procedure is adopted and the predicted and measured diameters are shown in Figure 4a. The colored markers and the solid lines denote the measured and predicted diameters. The measured diameter is very close to the predicted values, verifying the effectiveness of the identified linear models. Figure 4b shows the percentage deviation between the measured and predicted diameters. All the deviations are less than 4% and the deviations of the 0.25 mm nozzles is within 2%. It demonstrates that we can predict the diameter of hydrogel fiber according to the nozzle and the driving pressure.

### 3.2. Control on the Diameter of the Fabricated Hydrogel Fiber

In Section 3.1, we can use the identified linear models to predict the diameter of the fabricated hydrogel fiber according to the nozzle and the driving pressure. On the other hand, we can also use the linear models to control the diameter of the hydrogel fiber. Equation (1) can be rewritten as

(2) $$p=\frac{d-c}{k}$$

This equation can then be used in the diameter control of the hydrogel fibers. If we want to fabricate a hydrogel fiber with a designed diameter, we firstly select the nozzle according the comparison between the designed diameter and offset coefficient *c*. Subsequently, the driving pressure can be calculated via Equation (2) with the identified coefficients listed in Table 3. For example, if the designed diameter is 280 μm, the 0.25 mm nozzle will be chosen as the designed diameter is larger than the offset coefficient of the 0.25 mm nozzle (223.13 μm) and less than that of the 0.41 mm nozzle (402.82 μm). We assign 15 different diameters and the designed and measured diameters are recorded in Table 4. The errors between the measured and designed diameters are all within ±2.5%. In addition, if the measured diameter is plotted against the designed diameter, the experimental results in Table 4 can be graphically represented in Figure 1c. In this figure, the red line is the 45° line that represents a unitary mapping between the designed and measured diameters in the ideal case. The experimental results are plotted in blue dots. It is observed that the blue dots distribute very close to the 45° line, demonstrating that we can precisely control the diameter of the hydrogel fiber via Equation (2). This is helpful in the application of cell-laden hydrogel fibers in the 3D bio-fabrication of complex tissue structures.

### 3.3. The Cell Viability in the Hydrogel Fiber

During the fabrication process of the cell-laden hydrogel fibers, the cells are exposed to the CaCl_2_ solution. As the calcium ions are likely to impact the cell membrane, it is very important to experimentally investigate the damage of calcium ions to the cells in order to guarantee a satisfactory level of cell viability in the fabricated hydrogel fibers. In the cell viability experiments, we fabricate cell-laden hydrogel fibers in the configuration of (0.41, 2.2) and its diameter is measured to be 596.4 μm, very close to the predicted value of 588.5 μm.

After the cell-laden hydrogel fibers solidify in the CaCl_2_ solution, they are divided into four groups, and each group is soaked inside the Ca^2+^ bath for 0, 5, 10, and 15 min, respectively. Then the hydrogel fibers are collected from the bath and rinsed by PBS to remove the residual calcium ions. Finally, the cell-laden hydrogel fibers are transferred to four different Petri dishes with DMEM and cultured in the incubator with the appropriate environment.

**Figure 5:**
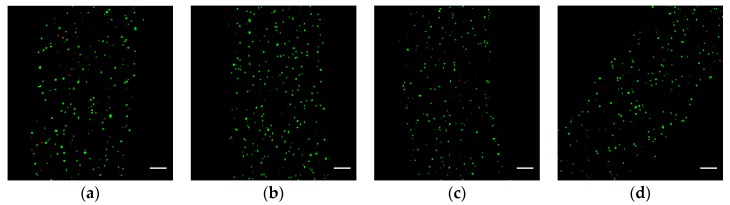
Fluorescence images showing the distribution of LIVE/DEAD cells in the hydrogel fibers after 24 h of culturing: (**a**–**d**) are the four samples soaked in the Ca^2+^ bath for 0, 5, 10, and 15 min, respectively. The green and red dots represent the live and dead cells, respectively; the scale bar is 100 μm.

After 24 h of culturing, each group is washed in PBS three times and then immersed into the fluorescent reagent solutions (FDA and PI as the markers of live and dead cells, respectively) for 5 min. With the help of an ultraviolet laser, we can identify the live and dead cells under the laser confocal microscopy. Figure 5 shows the superposition of all the fluorescent images at different layers. We find out that the cell viability is 83 ± 2% for each group through manually counting the live and dead cells in each image. The viability of cells encapsulated in all hydrogel fibers is similar to each other. The experimental result shows that the Ca^2+^ infiltrated into hydrogel fibers has little impact on the cells during the process of fabrication of cell-laden hydrogel fibers, even if the cell-laden hydrogel fibers are soaked in the Ca^2+^ bath for an additional 15 min. In the meantime, it is also a validation that the cells encapsulated in the hydrogel fiber are not seriously damaged during the fabrication process, which indicates that the method in the paper is feasible to fabricate cell-laden hydrogel fibers.

## 4. Discussion

A pneumatic micro-extrusion system is developed in this paper to fabricate sodium alginate hydrogel fibers. By adjusting the driving pressure, the sodium alginate solution can be extruded out through a tapered nozzle and falls into the CaCl_2_ solution. The alginate solution starts to solidify immediately after it contacts the Ca^2+^ bath owing to the crosslinking effect. By the use of a PID closed-loop control, a high precision of 0.01 PSI is achieved in the driving pressure control, thus enabling stable and fine adjustment of the driving pressure during experiments. Moreover, the cell viability in the experiments is more than 80%, which is comparable with other related research work. This indicates that the cells are not seriously damaged during the fabrication process of cell-laden hydrogel fibers.

The developed system provides a stable platform for the hydrogel fiber fabrication, and the diameter of the hydrogel fiber has a good uniformity. We fabricate four hydrogel fibers in four different configurations. The diameters at 10 different positions of each fiber are measured, and the standard deviations are all less than 3%. The hydrogel fibers fabricated in the repetitive experiments have a good uniformity in the diameter as well. Therefore, we can repeatedly obtain hydrogel fibers with the same diameter if we can control the configurations of the system, i.e., the nozzle size and the driving pressure. This is important to guarantee the geometric accuracy in the hydrogel fiber based 3D bio-fabrication applications.

Interestingly, experiment results show that a linear relationship can be found between the diameter of the hydrogel fiber and the driving pressure. Linear curve fitting is conducted to identify the coefficients of the above linear model. Then a series of experiments are carried out, and the measured diameters are compared with the predicted diameters. The deviations are all within 5%. This result demonstrates the effectiveness of the linear model. In addition, according to the fluid mechanics properties of fully developed flow in the round pipe, the diameter of fluid flow is proportional to the pressure drop between the inlet and the outlet. Our identified model can be treated as its extension to the high viscosity solution flows in the liquid.

Except for the diameter prediction, an important application of the identified linear model is the utilization of its inversion in the diameter control of the hydrogel fiber. We manually assigned 15 different diameters and the respective driving pressures are calculated using the inversion of the linear model. Then 15 hydrogel fibers are fabricated and the diameters are measured. Experimental results show that the differences between the designated and measured diameters are all within ±2.5%. This gives us the capability of directly controlling the diameter of the hydrogel fibers through the linear model.

It must be noted that there are some limitations in the identified linear model. For example, the driving pressure cannot be set to zero as the capillary force of the nozzle and the friction force will stop the alginate solution from falling. On the contrary, the driving pressure cannot be set to very large values as the alginate solution will spurt from the nozzle, which is unstable and uncontrollable.

Finally, the porcine fetal muscle cells are selected and encapsulated in the hydrogel fibers to validate the applicability of the developed system in the bio-fabrication. The cell-laden hydrogel fibers are soaked into the CaCl_2_ solution for four different periods of time after fabrication. Then they are cultured in appropriate environment and observed using a laser confocal microscopy. The cell viability of each sample is calculated to 83 ± 2% after 24 h of culturing, which is adequate for the cell proliferation. It is interesting that there is almost no difference in the cell viabilities among the samples, which indicates that the Ca^2+^ ions existing in the fabrication process have no impact on the cells. This is also a proof that the sodium alginate hydrogel fibers can provide cells with the support to survival and micro channels for the nutrition transport. This is of great significance for assembling cell-laden hydrogel fibers into 3D structure to obtain active tissues.

## 5. Conclusions

In the study, a pneumatic micro-extrusion system has been developed to fabricate cell-laden hydrogel fibers utilizing the crosslinking of sodium alginate with calcium ions. With the help of PID algorithm in the control, the driving pressure has a high precision and is more stable during the fabrication of the hydrogel fibers. Meanwhile, alginate hydrogel fibers have a good uniformity, which is an advantage in assembling them into complex structures. Furthermore, a series of experiments are carried out and a good linear relationship between the diameter of hydrogel fiber and the driving pressure is observed. We can predict or design the diameter of hydrogel fiber through the linear relationship. Experimental results show that the deviations between the measured and calculated diameters are within ±2.5%. In the cell viability test, the cells encapsulated in the hydrogel fiber have a viability of 83 ± 2%, which is significant for assembling cell-laden hydrogel fibers into complex tissues structure in the future.

## Figures and Tables

**Table 1:** All the measured diameters for the four hydrogel fibers (unit: μm).

(*D*, *P*)	1	2	3	4	5	6	7	8	9	10	Mean ± S.D.
(0.2, 2.0)	202.9	222.9	209.7	208.6	209.3	211.3	217.7	200.9	200.1	218.7	211.4 ± 8.3
(0.25, 1.5)	341.8	346.4	359.1	355.0	362.0	356.2	343.9	360.0	356.7	351.7	351.7 ± 8.3
(0.41, 1.5)	530.5	524.2	519.5	528.9	527.6	527.2	533.1	533.1	528.5	531.3	529.0 ± 7.8
(0.61, 1.0)	780.6	796.1	784.2	764.8	796.1	806.3	786.2	806.4	772.0	778.8	785.2 ± 14.8

**Table 2:** The statistics of the measured diameters in different configurations (unit: μm).

*D* (mm)	1.0 PSI	1.4 PSI	1.8 PSI	2.2 PSI	2.6 PSI	3.0 PSI
0.2	210.4 ± 3.3	219.2 ± 4.3	223.0 ± 2.5	215.9 ± 1.9	224.8 ± 4.4	231.3 ± 3.3
0.25	274.7 ± 3.2	302.0 ± 3.1	331.7 ± 3.2	352.6 ± 2.7	366.9 ± 5.2	390.0 ± 4.1
0.41	490.2 ± 4.6	516.3 ± 3.6	556.0 ± 3.1	583.2 ± 2.6	632.9 ± 7.7	651.0 ± 6.3
0.61	767.7 ± 4.5	814.5 ± 2.9	866.0 ± 6.5	908.6 ± 4.6	995.7 ± 5.8	1038.5 ± 5.9

**Table 3:** The identified coefficients of the linear models. *D* is the inner diameter of nozzle; *k* and *c* are the slope and offset of the linear relationship.

*D*	0.2 mm	0.25 mm	0.41 mm	0.61 mm
***k***	8.1747	56.596	84.386	138.59
***c***	204.42	223.13	402.82	621.35

**Table 4:** The comparison between the designed and measured diameters (unit: μm).

No.	1	2	3	4	5	6	7	8	9	10	11	12	13	14	15
Designed	280	310	340	370	400	450	500	550	600	650	800	850	900	950	1000
Measured	284.5	312.9	341.4	366.2	405.5	456.4	487.5	556.8	599.5	646.9	797.6	841.0	906.9	940.1	1005.5
Error (%)	1.62	0.93	0.42	−1.02	1.37	1.42	−2.49	1.24	−0.09	−0.48	−0.30	−1.05	0.76	−1.04	0.55

**Figure A1:**
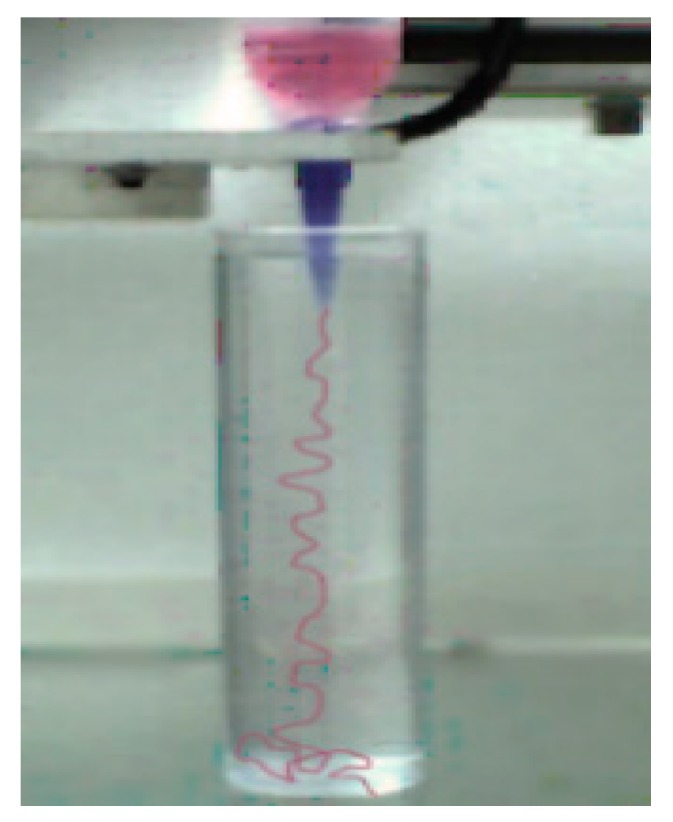
Formation of spiral curl of alginate hydrogel fiber.

## Supplementary Materials

The following is available online at www.mdpi.com/2072-666X/8/5/161/s1. Video S1: Formation of spiral curl of alginate hydrogel fiber.

## Appendix A

The Ca-alginate hydrogel fiber is successfully fabricated as shown in Figure A1. We use the 0.41 mm nozzle and the driving pressure is set to 2.0 PSI. For a clear visualization of alginate hydrogel fiber in the CaCl_2_ solution, we mix the red culture medium with the sodium alginate solution. During the fabrication process, the hydrogel fiber may form spiral curls. As the driving pressure increases, the phenomenon is more likely to occur (the Video S1 of the fabrication process in supplementary materials shows the behavior). This phenomenon is probably caused by the friction between the CaCl_2_ solution and the hydrogel fiber and the buoyance of the hydrogel fiber which slow down the falling of the hydrogel fiber into the CaCl_2_ solution. The spiral hydrogel fiber is undesirable in constructing more complex structures. We should place the nozzle vertically during the fabrication to avoid this phenomenon.
